# GPS collars have an apparent positive effect on the survival of a large carnivore

**DOI:** 10.1098/rsbl.2021.0128

**Published:** 2021-06-30

**Authors:** Cyril Milleret, Richard Bischof, Pierre Dupont, Henrik Brøseth, John Odden, Jenny Mattisson

**Affiliations:** ^1^ Faculty of Environmental Sciences and Natural Resource Management, Norwegian University of Life Sciences, 1432 Ås, Norway; ^2^ Norwegian Institute for Nature Research (NINA), 7485 Trondheim, Norway; ^3^ Norwegian Institute for Nature Research (NINA), 0855 Oslo, Norway

**Keywords:** population level, representativeness, population dynamics

## Abstract

Are instrumented animals representative of the population, given the potential bias caused by selective sampling and the influence of capture, handling and wearing bio-loggers? The answer is elusive owing to the challenges of obtaining comparable data from individuals with and without bio-loggers. Using non-invasive genetic data of a large carnivore, the wolverine (*Gulo gulo*) in Scandinavia, and an open-population spatial capture–recapture model, we found a 16 (credible interval: 4–30) percentage points lower mortality probability for GPS-collared individuals compared with individuals without GPS collars. While the risk of dying from legal culling was comparable for collared and non-collared wolverines, the former experienced lower probability of mortality due to causes other than legal culling. The aforementioned effect was pronounced despite a potentially lower age—and therefore likely higher natural mortality—of collared individuals. Reports of positive effects of bio-loggers on the survival of individuals are uncommon and we argue that GPS collars could shield animals from poaching. Our results highlight the challenges of drawing population-level inferences for populations subjected to poaching when using data from instrumented individuals.

## 1. Introduction

Telemetry and bio-logging systems have been crucial in expanding our understanding of the ecology and cryptic behaviour of wildlife. Data from instrumented animals are often the only available information from which to draw population-level inferences, forcing ecologists to make the assumption that instrumented animals are representative of the population as a whole [1]. There are two main reasons why this assumption may not hold: (a) instrumented animals are sampled non-randomly from the population, and (b) bio-logging and tracking themselves alter the biology of instrumented animals.

### (a) Non-random sampling

The selection of individuals in telemetry studies is rarely random [1]. For example, variation in the vulnerability to physical capture linked to biological attributes (e.g. behaviour) is bound to lead to biased conclusions if estimates are extrapolated to the population level.

### (b) Tag-effect

The methods that impact the study species will inadvertently introduce bias as the study system will be altered through the process of observation [2,3]. First, capturing and handling are both stressful for animals [4,5]. Second, although benign in most cases [6], attaching bio-logger devices can influence the behaviour or even have long-term detrimental effects on individuals [6,7]. Bio-logging can also have indirect consequences as bio-loggers can interact with management decisions and illegal actions [8], and lead to or prevent lethal events using the information from the logger [8,9]. This may be particularly acute for controversial species such as large carnivores.

Here, we used long-term non-invasive genetic sampling (NGS) data of a large carnivore, the wolverine (*Gulo gulo*) in Scandinavia, and compared survival probabilities of individuals with and without GPS collars using an open-population capture–recapture model (OPSCR). This unique dataset contained data from individuals without capturing them, as both instrumented and non-instrumented individuals were sampled during NGS.

## 2. Material and methods

### (a) GPS-collared individuals

Between 2010 and 2015, 43 (♀21; ♂22) wolverines were captured from helicopter [10] and equipped with GPS collars in the central and northern parts of Norway (electronic supplementary material, figure S1). The collars were fitted with a release mechanism that usually breaks apart after 0.5–2 years. After winter 2016/2017, no wolverine wore a GPS collar (see electronic supplementary material, appendix S1 for further details).

### (b) Non-invasive genetic sampling

The Scandinavian wolverine population is monitored annually by Norwegian and Swedish authorities using NGS from scats, urine and shed hairs [11]. NGS targets all individuals more than or equal to 1 year old, including GPS-collared individuals. Genetic analyses of hair or blood samples from collared individuals allowed us to match them with the NGS dataset. As we aimed to compare the survival of wolverines with and without GPS collars using NGS, we only retained NGS data collected within 70 km (greater than 7*σ*, see definition below) of all collected samples from collared individuals to ensure that we also obtained detections from individuals with home ranges in the vicinity of the collared individuals. This resulted in 4989 (♀2446; ♂2543) non-invasive genetically identified samples from 1036 (♀555; ♂481) individuals collected over eight consecutive monitoring seasons (December–June) between 2009/2010 and 2016/2017 in two non-adjacent regions (central and northern Norway; electronic supplementary material, figure S1 and table S1). In addition, we obtained recovery locations and genetic identification data from all 424 (♀219; ♂205) legally culled individuals (authorized by management authorities, and motivated by sheep and semi-domestic reindeer depredation, electronic supplementary material, appendix S1), and 11 (♀6; ♂5) individuals dead owing to other reasons (i.e. 4, unknown; 2, verified poaching; 4, car collision; 1, disease).

### (c) Open-population capture–recapture model

To estimate survival probabilities of wolverines from NGS, we built a Bayesian hierarchical state-space OPSCR model composed of three submodels for (i) population dynamics, (ii) density and movements, and (iii) detection during DNA searches [11–15].

#### (i) The population dynamics model

We used a multistate formulation [15,16] where each individual life history was represented by a succession of four discrete states *z_i_*_,*t*_: (i) ‘unborn’ if the individual has not been recruited in the population; (ii) ‘alive’; (iii) ‘dead legal’ if it has died from legal culling between the start of the previous and current monitoring seasons; or (iv) ‘dead’ if it has died from any other cause between the start of the previous and current monitoring seasons, or died earlier, regardless of the cause. We used data augmentation, whereby additional, undetected individuals are available for inclusion in the population at each time step [17,18].

During the first year, individuals are designated as ‘unborn’ or ‘alive’ so that *z*_*i*,1_ ∼ dcat(1−ψ, ψ, 0, 0), where ψ represents the probability to be part of the population at *t* = 1.

For *t* ≥ 2, *z_i_*_,*t*_ is conditional on the state of individual *i* at *t* − 1:

— If *z_i_*_,*t*−1_ = 1, individual *i* can be recruited (transition to state 2) with probability $\gamma_{t\;}$, so *z_i_*_,*t*_ ∼ dcat($1-\gamma_{t},\gamma_{t},0,0)$.
— If *z_i_*_,*t*−1_ = 2, individual *i* can survive and remain *z_i,t_* = 2 with probability *Φ_t_*, die from culling and transition to *z_i_*_,*t*_ = 3 with probability *h_t_*_,_ or die from other causes and transition to *z_i_*_,*t*_ = 4 with probability *w_t_*, so that *z_i_*_,*t*_ ∼ dcat(0, *Φ_t_*, *h_t_*, *w_t_*), where *Φ_t_* = 1 − *h_t_* − *w_t_*_._ All legal culling mortality events were reported, but most other mortality remains cryptic. Imperfect detection of non-culling mortality prevents further breakdown of estimates by cause-specific mortality, such as natural, traffic and poaching deaths.
— All individuals in dead states (*z_i_*_,*t*−1_ = 3 or 4) transition to *z_i_*_,*t*−1_ = 4, the absorbing state, with probability 1.

We created a binary covariate (GPS*_i,t_*) with value 1 if the individual *i* was wearing a GPS collar at any time during the monitoring season *t*, and 0 otherwise. To quantify differences in culling $\left(\beta h_{\mathrm{GPS}}\right)$ and other $\left(\beta w_{\mathrm{GPS}}\right)$ mortality probabilities between collared and non-collared individuals, we expressed mortality probabilities as

2.1 $$\begin{array}{c} \mathrm{logit}(w_{i,t})\;=\;w0_{t}\;+\;\beta w_{\mathrm{GPS}}\;\times \;{\mathrm{GPS}}_{i,t}\\ \mathrm{logit}(h_{i,t})\;=\;h0_{t}\;+\;\beta h_{\mathrm{GPS}}\;\times \;{\mathrm{GPS}}_{i,t} \end{array}\},$$

where $w0_{t}$ and $h0_{t}\;$ are the year-specific mortality probabilities of non-collared individuals.

#### (ii) The movement model

We used an inhomogeneous point process to model the distribution of individual activity centres (ACs) with a spatial intensity λ(s) (where ***s*** is a vector of spatial coordinates of ACs) [19]. We discretized the habitat into a grid of 20 × 20 km habitat cells to allow the placement of individual AC $s_{i,t}$ as a function of a spatial covariate (*X*). The initial individual AC locations $s_{i,1}$ were conditional on *X*:

2.2 $$\lambda (s_{i,1})\;=\;e^{B_{\mathrm{Dens}}X(s_{i,1})},$$

where $X(s_{i,1})$ is the value of the spatial covariate at $s_{i,1}$ and $B_{\mathrm{Dens}}$ the slope parameter describing the relationship between the habitat covariate and density. We defined *X* as the average number of known wolverine dens as a proxy for wolverine density (electronic supplementary material, figure S8; [11]). For *t* > 1, the probability density of $s_{i,t}$ was conditional on the spatial covariate *X* and the Euclidean distance to $s_{i,t-1}$:

2.3 $$\lambda (s_{i,t}|s_{i,t-1},\;\tau )\propto \;e^{-\frac{\left||s_{i,t}-s_{i,t-1}^{2}|\right|}{2\tau^{2}}}\times e^{B_{\mathrm{Dens}}X(s_{i,t})},$$

where *τ* is the standard deviation of a bivariate normal distribution centred on $s_{i,t-1}$. Under this specification, movement is described as an isotropic Gaussian random walk weighted by the spatial covariate *X* [11,13,19], and *τ* regulates the distance that individuals are likely to move between years. Such a movement feature can help distinguish between mortality and emigration [13,20].

#### (iii) The observation model

We used the half-normal function to model detection probability, whereby the probability *p* of detecting individual *i* at detector *j* and time *t* decreases with distance (*D_i,j,t_*) between the detector and the AC (*s_i,t_*):

2.4 $$p_{i,j,t}=p_{0_{i,j,t}}\exp\left(\frac{-D_{i,j,t}^{2}}{2\sigma^{2}}\right),$$

where *p*_0_ is the baseline detection probability, and *σ* the scale parameter.

To account for individual, spatial and temporal heterogeneity in detection probability, we included several linear effects on a logistic scale on the baseline detection probability (*p*_0_) to account for search effort (length of GPS search tracks, *β*_Tracks_), accessibility (distance from the nearest road, *β*_Roads_), snow cover (*β*_Snow_), previous detections of individuals (*β*_PrevDetections_), and whether the individual was wearing a GPS collar (*β*_GPS_) or not. Because NGS can be country- and county-specific, we also estimated yearly baseline detection probabilities ($p_{0\mathrm{Intercept}}$) for each county (see further details in electronic supplementary material, appendix S1).

To decrease the number of detectors involved in the calculation of *p_i,j,t_* and, therefore, the computation burden, we assigned the location of detections to the closest detector defined as the cell centre of a 10 × 10 km detector grid. The detector grid cells were further subdivided into 25 subdetectors (2 km resolution) [21], and each detection was assigned to the closest subgrid. We then modelled the frequency of subdetectors with greater than or equal to 1 detection *y_i,j,t_* as a binomial response with sample size *K_j_*, the number of subdetectors in grid cell *j* that overlapped with the habitat [21]:

2.5 $$y_{i,j,t}\;∼\mathrm{Binomial}(p_{i,j,t}\;\times \;I(z_{i,t}\;=\;2),K_{j}),$$

where $I(z_{i,t}\;=\;2)$ is an indicator function used to condition detection on the individual being alive. This design allowed us to reduce the number of detectors *j* involved in the calculation of $p_{i,j,t}$ while retaining as many binary detections as possible [21]. In addition, we added a 60 km buffer (greater than 6σ, [22]) around the detector grid to allow the placement of AC, and, therefore, the movement of individuals in and out of the trapping grid [11,12].

### (d) Parameter estimation

To account for confounding factors, all model parameters were region- and sex-specific, except for the effects of the collar $\left(\beta w_{\mathrm{GPS}},\;\beta h_{\mathrm{GPS}}\;\mathrm{and}\;\beta_{\mathrm{GPS}}\right)$ which were assumed identical for both regions and sexes (owing to sample size limitations). Because age was not known for individuals detected with NGS, we could not account for its potential effect on parameters in the OPSCR model. We fitted the Bayesian OPSCR model using Markov chain Monte Carlo (MCMC) simulation with NIMBLE [23,24] in R v. 3.3.3 [25]. We used the local evaluation approach [26] to increase MCMC efficiency (nimbleSCR [27,28]). We ran four chains, each with 42 500 iterations including a 12 500-iteration burn-in. We considered the model as converged when the Gelman–Rubin diagnostic (Rhat, [29]) was less than 1.1 for all parameters and by visually inspecting the trace plots. In addition to providing estimates of the coefficients $\beta w_{\mathrm{GPS}}$ and $\beta h_{\mathrm{GPS}}$, we also calculated the median expected mortality probabilities for individuals with and without GPS collars and computed the percentage point difference and its associated 95% credible interval (CrI) using the posterior distribution.

## 3. Results

**Figure 1.**
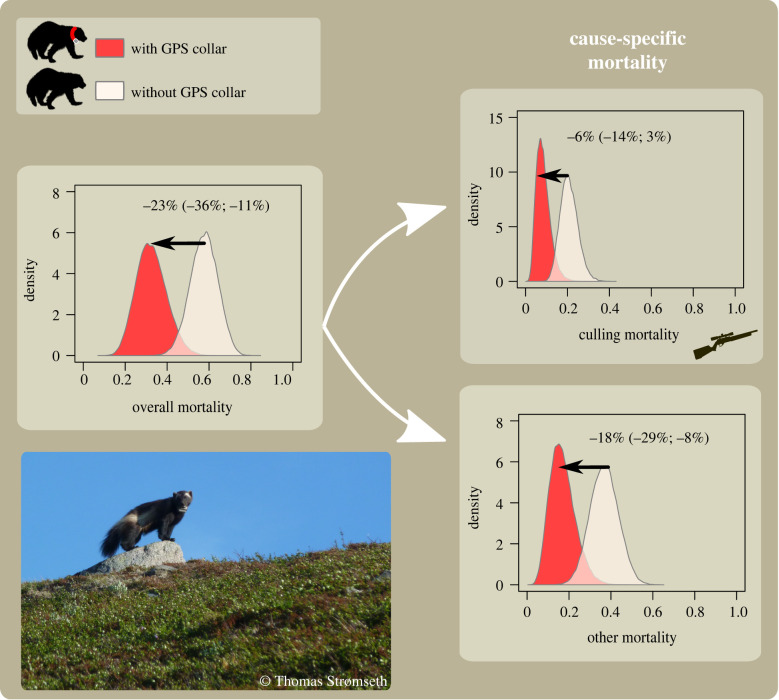
Posterior distributions of mortality probabilities for male wolverines with and without a GPS collar in the northern study area between 2010/2011 and 2011/2012. Estimates were obtained using a Bayesian open-population spatial capture–recapture model and NGS data. Expected percentage point difference (and associated 95% credible interval) in mortality probabilities between individuals with and without collars are shown above the arrows indicating the direction of reduction in risk. This example only displays estimates for male wolverines from the northern study area; differences in mortality probabilities depended on the baseline probabilities (*h*_0_, *w*_0_) and varied with year, sex and regions because the effect of GPS collars on mortality rates ($\beta w_{\mathrm{GPS}}$, $\beta h_{\mathrm{GPS}}$) was quantified on the logit scale (equation (2.1)).

GPS-collared wolverines had a 16 percentage points (95% CrI: (4; 30 percentage points)) lower overall mortality probability (median = 19%; (7%; 43%)), compared with non-collared individuals (median = 35%; (13%; 66%); e.g. males in the northern area, figure 1). This difference was attributable mainly to lower probability of mortality due to causes other than legal culling ($\beta w_{\mathrm{GPS}}$ = −1.08 (−1.86; −0.46)), and to a lesser extent to a lower probability of mortality due to legal culling ($\beta h_{\mathrm{GPS}}$ = −0.37 (−1.09; 0.25); figure 1). Collared individuals had a 13 percentage points (2; 26 percentage points) lower probability to die from causes other than legal culling (12% (2%; 29%)), compared with individuals without a GPS collar (25% (4%; 48%)). Collared individuals had a 3 percentage points (−2; 11 percentage points) lower probability to die as a result of legal culling (7% (2%; 27%)), compared with individuals without a GPS collar (10% (2%; 33%)). Additional results and sex- and region-specific estimates are presented in electronic supplementary material, appendix S2 and figures S2–S7.

## 4. Discussion

We found that GPS-collared wolverines had a lower mortality probability than individuals without a collar, mainly due to causes other than legal culling. There are three main, non-mutually exclusive, explanations for this result: (i) mortality probability of wolverines selected for GPS collaring differed from the population average (non-random sample), (ii) instrumentation altered wolverine mortality (tag-effect), and (iii) the NGS and OPSCR approach inadvertently introduced a bias (analytical artefact). Being observational in nature, our study does not allow us to isolate an explanation unequivocally. However, we argue that the tag-effect could be the primary cause for the observed difference in mortality between instrumented and non-instrumented animals.

### (a) Non-random sampling

NGS and the collaring of wolverines targeted all segments of the population (except cubs of the year). Captures from helicopter did not target specific individuals by following any fresh wolverine tracks encountered in snow but may have been unintentionally biased towards individuals more vulnerable to capture once detected (e.g. subadult individuals). The OPSCR model did not include age as it was not available for individuals detected solely with NGS. This means that we were unable to distinguish between adult and subadult mortality (the latter being usually higher). This could explain the relatively high observed mortality estimates compared with those reported previously [30]. However, the proportion of 1-year-olds among collared individuals (41%) was higher than their expected prevalence in the population (29%; [31]). Given that younger wolverines typically have a lower survival [30], we would expect higher mortality estimates for collared animals than the population average. Yet, we detected the opposite effect, which suggests that the explanation for the mortality difference lies elsewhere.

### (b) Tag-effect

Wearing a GPS collar itself impacts the mortality of instrumented wolverines. Other causes of mortality include natural (e.g. age, diseases, starvation, intra- and interspecific killing), traffic and illegal killing, which we cannot differentiate. The literature generally reports negative or neutral effect of wearing bio-loggers on the survival of individuals [6,32], and we are not aware of any mechanism that could give a competitive advantage to GPS-collared wolverines that would decrease other causes of mortality. While punishments for illegal killing can be severe (e.g. in Norway [33]), poaching accounts for a large portion of carnivore mortality in Scandinavia [30,34–37]. A plausible explanation for the observed result is that GPS collars shield individuals against illegal killing. First, the collars themselves can act as a deterrent as the chance of detecting poaching events increases. Second, collared wolverines were captured as part of a wildlife–human conflict project studying predation on semi-domestic reindeer and sheep (e.g. [38]). In Norway, compensation for livestock losses to carnivores is estimated based on the number of detected domestic prey that can be documented as killed by a carnivore. As information provided by GPS collars was partly used to conduct predation studies, it generally resulted in a higher number of documented kills, which could give an incentive to keep GPS-collared individuals alive [37].

### (c) Analytical artefact

We cannot exclude the presence of analytical artefact, especially since robust goodness of fit tests for Bayesian OPSCR models are not yet available ([39]; electronic supplementary material, appendix S1). However, to avoid comparing different parts of the population, we ensured overlapping spatial (electronic supplementary material, appendix S2, and figure S1) and temporal extents between collared and non-collared individuals, while estimating sex- and region-specific parameters (electronic supplementary material, appendix S2 and figures S4–S7). The OPSCR model also accounted for important sources of individual (sex, previous detections), temporal (year) and spatial heterogeneity (snow cover, accessibility, search effort) in detectability ($p_{0}$; electronic supplementary material, appendix S2 and figures S4–S7), including differences in detectability between GPS-collared and non-collared individuals (*β*_GPS_, electronic supplementary material, figure S7). The lower detectability of GPS-collared individuals (*β*_GPS*,*_ electronic supplementary material, figure S7) could be due to the relatively higher proportion of young individuals among GPS-collared individuals compared with individuals detected with NGS, which usually have a lower detectability than adult territorial individuals [40].

It has previously been suggested that instrumented and non-instrumented individuals have different survival probabilities in other large carnivore populations [41–43], but to our knowledge, this is the first study to compare survival probabilities of carnivores with and without GPS collars, using an independent source of data collected at the individual level. Our finding that bio-loggers have a positive effect on survival is rarely reported and has important implication for management and conservation. This is especially so if the positive effect is caused by collared animals being shielded from poaching. Indeed, many studies use data from instrumented individuals to draw inferences about populations subjected to poaching [34,35,37].

## Data accessibility

Data available from https://github.com/Cyril-Milleret/Public/tree/master/WolverineGPSSurvival.
